# Bone Cement Waste in Total Knee Arthroplasty: A Preliminary Study of Scope and Cost

**DOI:** 10.1016/j.artd.2023.101162

**Published:** 2023-07-24

**Authors:** Geoffrey S. Tompkins, Kevin K. Howe, Rebecca Valderrama, Katie Sypher, Michael Griffin, Shih Ting Chiu

**Affiliations:** a Redwood Orthopaedic Surgery Associates, Santa Rosa, CA, USA; b Providence Health Orthopaedic and Sports Medicine Institute, Renton, WA, USA; c Providence Health Care, Portland, OR, USA

**Keywords:** Bone cement, Total knee arthroplasty, Methacrylate, Waste

## Abstract

**Background:**

Medical waste is both costly and detrimental to the environment, and operating room waste represents a substantial portion of this. To the authors’ knowledge, bone cement waste in total knee arthroplasty (TKA) has not previously been studied. The vast majority of TKA are cemented, and the volume of TKA is forecast to increase. Given this, we studied the waste resulting from the routine use of 2 40-gram bags of polymethyl methacrylate (PMMA) powder during cementing in primary TKA.

**Methods:**

We first studied the yield of commercially available plain and gentamicin medium-viscosity bone cement powder and calculated the cost/gram of product. We then collected the PMMA remaining after primary TKA to determine the average amount of waste, its cost, and possible correlations with patient and implant metrics that could improve efficiency and reduce waste of PMMA.

**Results:**

Overall, PMMA waste averaged 59% per TKA, at a median cost of $129 per case. Cost of waste was greater when gentamicin cement was used, as its cost was 2.5X that of plain cement. Implant sizes and surface area ranges were identified that could reliably allow the use of a single 40-gram package of powder, potentially reducing PMMA waste.

**Conclusions:**

While it is acknowledged that zero-waste cementing is not practical, any reduction in waste that does not compromise either the flow of surgery or the adequacy of fixation would be beneficial. Reevaluation of PMMA techniques could reduce waste, resulting in both cost savings and improved sustainability in arthroplasty.

## Introduction

Total knee arthroplasty (TKA) is the gold standard surgical treatment for end-stage degenerative joint disease of the knee. More than 600,000 TKAs are performed annually in the United States alone, and one study has forecast an increase to 935,000 annually by 2030 [1].

Although the use of cementless TKA implants has recently increased, the vast majority of TKAs are cemented [[2], [3], [4], [5]]. The American Joint Replacement Registry reported cemented TKA accounted for nearly 80% of TKA in the United States in 2021 [2], and other countries have reported even higher frequencies of cemented TKA [[3], [4], [5]].

A common practice in many operating rooms is to prepare 2 40-gram packages (“bags”) of cement per TKA, regardless of patient or implant size [6]. Excess cement is discarded, and the waste is most often sent to the landfill [7]. The purposes of this study were: 1) to quantitate the waste of polymethyl methacrylate (PMMA) in primary TKA; 2) to assess cost implications and scale these to projected volumes of TKA; and 3) to seek correlations that may predict PMMA requirements, potentially reducing PMMA waste.

## Material and methods

Institutional review board approval (Providence Health) and exemption (Sutter Health) were obtained.

### Preliminary PMMA yield measurement

A preliminary measurement of PMMA yield was performed at 64°F, preparing the same commercial packages of cement in mixers identical to those used in the operating room. The cured PMMA was weighed. Yield was determined for:

• 2 × 40 gram (g) bags of plain medium-viscosity (MV) (DePuy SmartSet MV, Depuy Synthes, Warsaw, Indiana) powder plus 2 × 18.88 g ampules of liquid monomer
• 1 × 40 g + 1 × 20 g bag of plain MV (DePuy SmartSet MV, Depuy Synthes, Warsaw, Indiana) powder plus 1 × 18.88 g ampule +1 × 9.44 g ampule liquid monomer
• 2 × 40 g bags of gentamicin-MV (SmartSet GMV, DePuy Synthes, Warsaw, Indiana) powder plus 2 × 18.88 g liquid monomer

### PMMA cost data

The contracted prices for plain and gentamicin-impregnated PMMA for the 2 health systems involved in this study were combined, and the mean value was determined.

### Calculations

The measured mass of cement waste (“PMMA waste”) was subtracted from the mean yield of the identical cement (2 × 40 g plain or gentamicin) to determine “PMMA used.” PMMA waste was divided by the mean yield of identical cement to determine “PMMA waste percentage.” PMMA waste was multiplied by the calculated cost/gram of identical cement to determine “PMMA waste cost.”

### Patient selection

Patients were prospectively enrolled. Any patient undergoing standard primary TKA for diagnoses other than trauma or tumor was considered eligible. Revisions and complex primary cases requiring stemmed components and/or augments were excluded.

### Patient data

Patient data collected were age, sex, height, and weight. Implant sizes (femoral, tibial, and patellar) were recorded. Manufacturer specifications were used to calculate the total surface area of the cemented portion of the implants.

### Surgical technique

Surgeries were performed through a medial parapatellar approach, utilizing a tourniquet, by 2 fellowship-trained arthroplasty surgeons. The same implant (Attune Posterior-Stabilized, DePuy, Warsaw, IN) and cement (DePuy SmartSet MV and SmartSet GMV) were used in all cases. The patella was resurfaced in all cases.

For each case, two 40 g bags of powder and two 18.88 g vials of liquid polymer were vacuum-mixed (Revolution, Stryker, Kalamazoo, MI) and applied using a cement gun. Plain or gentamicin cement was used at the surgeon’s discretion. Cement technique was the same for both surgeons: cement in the doughy phase was applied to the undersurface of the tibial component and the posterior condyles and posterior chamfers of the femoral component. Cement was also applied to all exposed femoral, tibial, and patellar surfaces, and the pilot hole for the tibial cone was filled with cement. Applied cement was manually pressurized.

All implants were seated at one time using the specifically designed impactors and compressors for each component. Extruded cement was removed in the usual fashion. All removed cement, as well as all unused cement remaining in the cement gun cannister and tubing, was collected. At the conclusion of the procedure, the cured waste cement was weighed, determining PMMA waste.

### Relationship between PMMA used and patient/implant metrics

PMMA used was plotted against patient height, patient weight, sum of [femoral + tibial] implant sizes (“fem + tib”-a proxy for cemented surface area), and total cemented surface area of implants (“surface area”) to identify correlations that could be useful for predicting the PMMA required for individual patients. In addition, 95% confidence limits (CLM) were determined for fem + tib and surface area vs PMMA used.

### Statistical methods

Patient demographics, implant measurements, and waste cost were summarized as frequency and percentage for categorical data; mean and standard deviation (SD) for normal distributed data; or median and interquartile range (Q1, Q3) for nonnormal distributed data, as appropriate. To evaluate the difference between MDs, *P*-values were generated by the chi-squared test for categorical variables, and the t-test or Wilcoxon 2 sample test used for continuous variables for comparison.

PMMA waste cost was further stratified and summarized by surgeon as well as plain PMMA cases vs gentamicin-PMMA for comparison. Correlation between patient/implant metrics and PMMA used was evaluated by Pearson correlation coefficients with *P*-values indicating the significance of correlation. Scatter plots to visualize the relationships are presented. SAS 9.4 (SAS Institute, Cary, NC) was used to process the data and perform the statistical analysis, and R 4.2.2 was used to create the figures.

## Results

### PMMA yield and cost

Mean (±SD) yield of PMMA (Table 1)

**Table 1:** PMMA yield/cost data.

2 × 40-gram gent		Price/gram
Mean yield (SD)	109.gm (1.4)	$294/109.6 = $2.68/gm Abx
2 × 40-gram plain		Price/gram
Mean yield (SD)	106.7 g (1.2)	$100/106.7 = $0.94/gm plain
1 × 40-g + 1 × 20-gram plain		Price/gram
Mean yield (SD)	81.9 g (0.7)	$85/81.9 = $1.04/gm plain
		(est.) $272/81.9 = $3.32/gm Abx

2 × 40 g bags of plain MV powder plus monomer (N = 5): 106.7 g (±1.15)

2 × 40 g bags of gentamicin-MV powder plus monomer (N = 5): 109.6 g (±1.4)

1 × 40 g bag +1 × 20 g bag plain MV powder plus monomer (N = 3): 81.9 g (±0.7)

Costs for MV powder plus liquid monomer

40 g gentamicin: $147 USD
20 g gentamicin: $125
40 g plain: $50
20 g plain: $ 35
20 g gentamicin: not available for study

Calculated costs/gram of PMMA

2 × 40 g gentamicin: $2.68/g
2 × 40 g plain: $0.94/g
1 × 40 g plain +1 × 20 g plain: $1.04/g

### Demographics and implant measurements

Forty five consecutive TKAs were included. One case was eliminated when it was realized the cannister had been discarded postoperatively without removing all cement. The remaining 44 patients were studied. Seventeen cases were performed by MD 1 and 24 by MD 2. Twenty patients were men and 24 were women. For the entire cohort, mean (±SD) age was 70 (±7), mean height 67 inches (±5), and mean weight 200 pounds (±46). Range of femoral implant sizes was 4-9 (median [Q1, Q3] 6 [5, 7]) and range of tibial sizes was 3-9 (median [Q1, Q3] 5 [5, 7]). Patellar implant sizes were 35 mm-41 mm (median [Q1, Q3] 38 [35, 38]). Mean surface area was 135.6 cm^2^ (±17).

There were no significant differences in patient or implant measurements between MD 1 and MD 2. Use of gentamicin-PMMA was significantly greater for MD 2 (100%) than MD 1 (6%) *P* < .0001 (Table 2).

**Table 2:** Demographics and implant measurements.

Variable		Entire cohort N = 44	MD1 17 (39%)	MD2 27 (61%)	*P*-values
Women	N (%)	24 (55%)	10 (59%)	14 (52%)	.6511
Men	N (%)	20 (45%)	7 (41%)	13 (48%)	
Plain PMMA	N (%)	16 (36%)	16 (94%)	0 (0%)	<.0001
Gentamicin-antibiotic PMMA	N (%)	28 (64%)	1 (6%)	27 (100%)	
Age	Mean (SD)	70 (7)	71 (10)	69 (6)	.5401
Height (inches)	Mean (SD)	67 (5)	68 (4)	67 (5)	.7253
Weight (lbs)	Mean (SD)	200 (46)	188 (39)	208 (48)	.1611
Femur size	Median (Q1, Q3)	6 (5, 7)	5 (6, 7)	7 (5, 6)	.2874
Tibia size	Median (Q1, Q3)	5 (5, 7)	4 (6, 7)	7 (5, 6)	.8357
Femur + tibia size	Median (Q1, Q3)	11.5 (10, 14)	10.0 (11, 14)	14.0 (10, 11)	.6996
Patella	Median (Q1, Q3)	38 (35, 38)	38 (35, 38)	38 (35, 38)	.8265
Total surface area (cm^2^)	Mean (SD)	134.6 (120)	130.6 (119)	137.5 (121)	.5429

### PMMA use and waste

Median PMMA used was 42 g ([Q1, Q3] [30, 53]), and median PMMA waste was 64 g ([Q1, Q3] [54, 77]). Median PMMA waste percentage for the cohort was 59% [Q1, Q3] [49, 72]) per case.

There were significant differences in PMMA waste between the 2 surgeons. Median PMMA waste for MD 1 was 79 g ([Q1, Q3] [76, 80]), and for MD 2 was 56 g ([Q1, Q3] [49, 65]) (*P* < .0001). Median PMMA waste percentage for MD 1 was 74% ([Q1, Q3] [71, 75]) and for MD 2 was 51% ([Q1, Q3] [45, 59]) *P* < .0001 (Table 3).

**Table 3:** PMMA calculated mass used, PMMA waste mass, waste cost, and PMMA waste mass percentage.

Measurement		Entire cohort	MD 1	MD 2	*P*-value
PMMA waste mass (g)	Median (Q1, Q3)	64 (54.77)	79 (76.80)	56 (49.65)	<.0001
PMMA used	Median (Q1, Q3)	42 (30.53)	28 (27.31)	51 (39.60)	<.0001
Waste %	Median (Q1, Q3)	59 (49.72)	74 (71.75)	51 (45.59)	<.0001

### Cost of PMMA waste

Overall, median PMMA waste cost was $129/case ([Q1, Q3] [75, 164]). Median PMMA waste cost for gentamicin cement was $154/case ([Q1, Q3] [131, 175]) and for plain cement $74/case ([Q1, Q3] [72, 75]) *P* < .0001. Median PMMA waste cost for MD 1 was $75 ([Q1, Q3] [72, 76]) and for MD 2 was $151 ([Q1, Q3] [131, 174]) *P* < .0001 (Table 4).

**Table 4:** PMMA waste cost ($) by MD and plain vs gentamicin.

PMMA waste cost		Entire cohort N = 44	Plain PMMA cases 16(36%)	Gentamicin-PMMA 28 (68%)	*P*-value
PMMA waste cost ($)	Median (Q1, Q3)	129 (75,164)	74 (72.75)	154 (131,175)	<.0001
		Entire cohort N = 44	MD1 17 (39%)	MD2 27 (61%)	*P*-values
PMMA waste cost ($)	Median (Q1, Q3)	129 (75,164)	75 (72.76)	151 (131,174)	<.0001

### Correlation between patient/implant metrics and PMMA used

**Figure 1:**
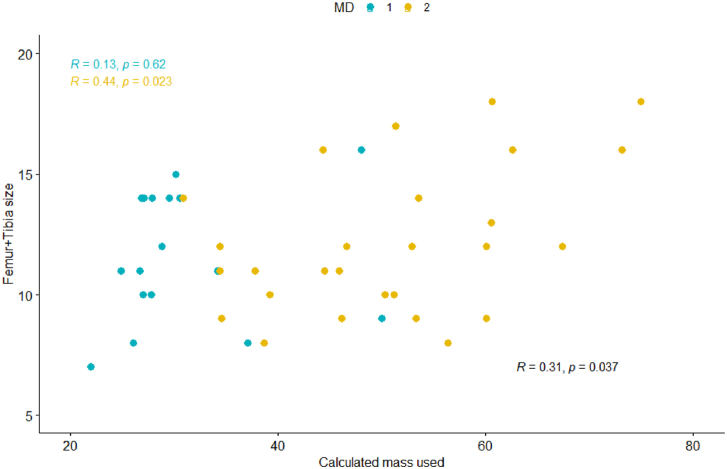
PMMA used vs sum of [femoral + tibial sizes] cohort: Scatter plot shows a weak linear correlation (R = 0.31, *P* = .037) MDs: Scatter plot shows no linear correlation for MD 1 and weak-moderate linear correlation for MD 2 (MD 1: R = 0.13, *P* = .62; MD 2 R = 0.44, *P* = .023).

**Figure 2:**
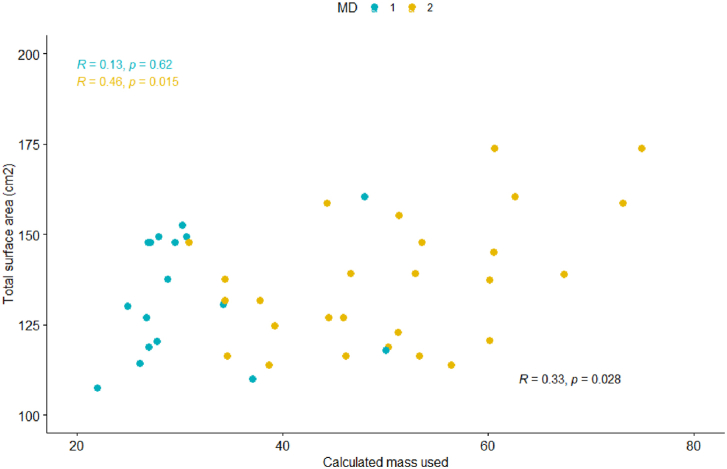
PMMA used vs total cemented surface area (cm^2^) cohort: Scatter plot shows a weak linear correlation (R = 0.33, *P* = .028) MDs: Scatter plot shows no linear correlation for MD 1 and weak-moderate correlation for MD 2 (MD 1: R = 0.13, *P* = .62; MD 2: R = 0.46, *P* = .015).

**Figure 3:**
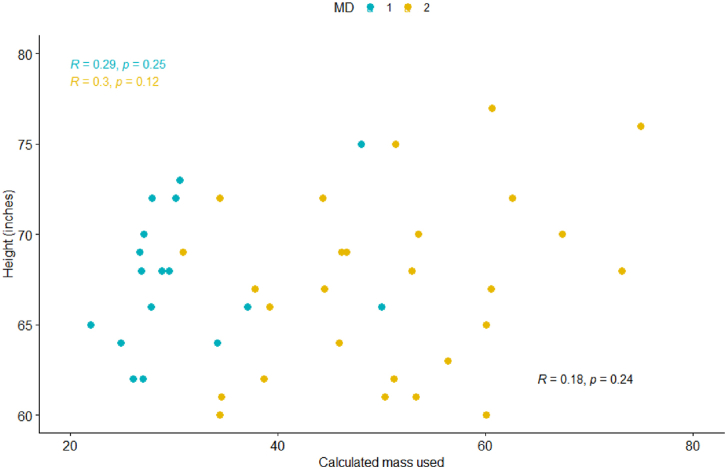
PMMA used vs patient height (in.) cohort: Scatter plot shows no linear correlation (R = 0.18, *P* = .24) MDs: Scatter plot shows weak linear correlations for both (MD1 R = 0.29, *P* = .25; MD 2 R = 0.3, *P* = .12).

**Figure 4:**
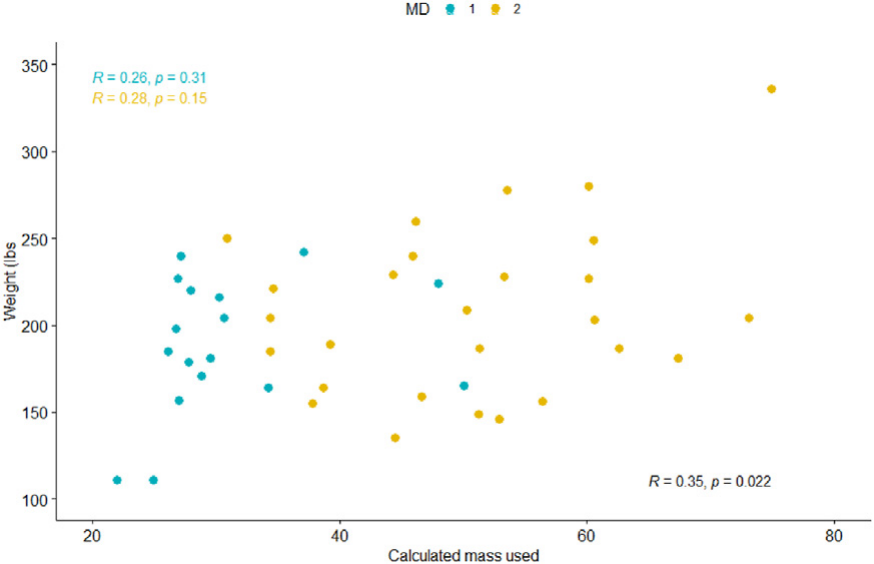
PMMA used vs patient weight (lbs.) cohort: Scatter plot shows weak linear correlation (R = 0.35, *P* = .022) MDs: Scatter plot shows weak linear correlation for both (MD 1:R = 0.26, *P* = .31; MD 2: R = 0.28, *P* = .15).

For the entire cohort (Figure 1, Figure 2, Figure 3, Figure 4), Pearson coefficients showed weak linear correlations between PMMA used and fem + tib (Fig. 1); surface area (Fig. 2); and patient weight (Fig. 4).

No correlation was observed between PMMA used and patient height (Fig. 3).

Because of the differences in PMMA used and PMMA waste between the 2 surgeons, Pearson correlations were also evaluated by individual surgeon. No or weak linear correlations were observed for each MD in each category (Figure 1, Figure 2, Figure 3, Figure 4).

**Figure 5:**
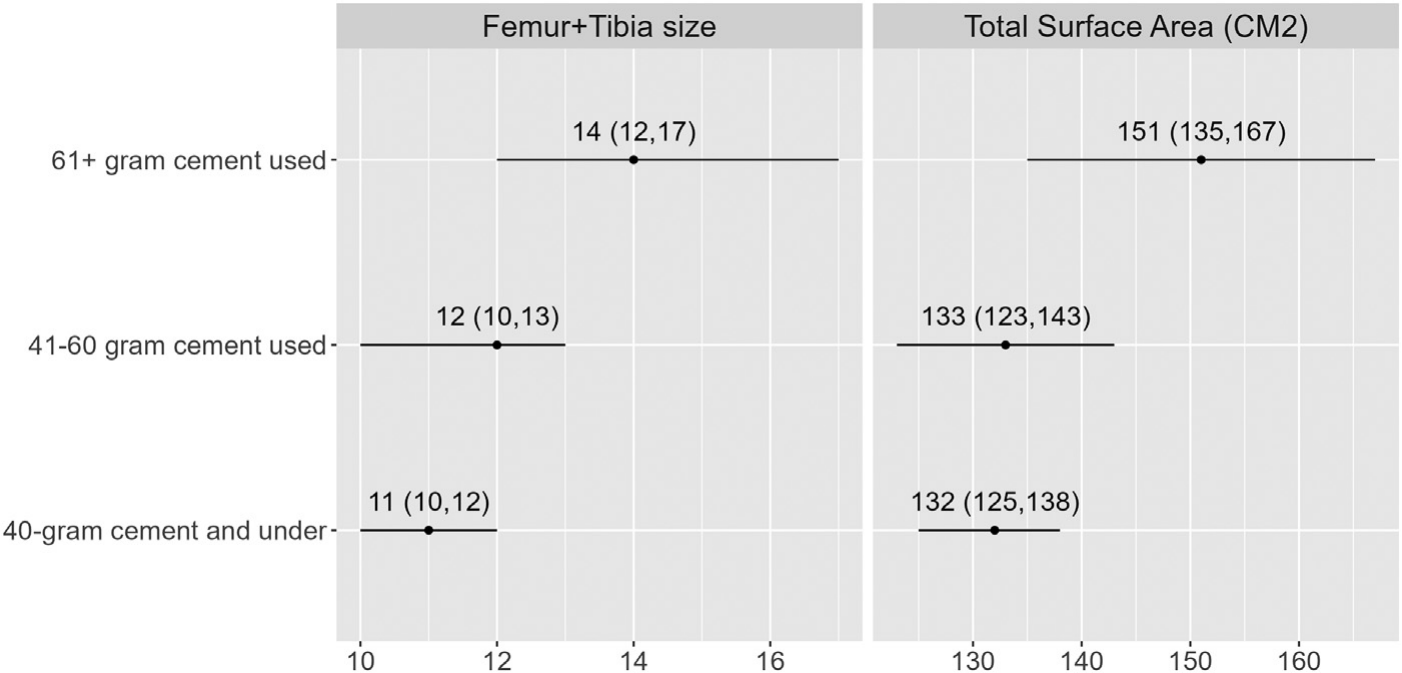
PMMA used vs sum of implant sizes and total cemented surface area 95% confidence limits.

While low *linear* correlations were observed, clusters of PMMA used were observed to correlate with ranges of fem + tib and surface area. For instance, in 100% of cases where fem + tib ≤8 (surface area ≤144.4 cm^2^), PMMA used was ≤40g (Table 5). In addition, for PMMA used ≤40-g, the mean fem + tib was 11 (95% CLM 10-12) and mean surface area was 132 cm^2^ (95% CLM 125-138) (Fig. 5).

**Table 5:** 100% Observations of implant size/surface area vs PMMA used.

PMMA used ≤40 grams	
Sum of femoral + tibial sizes ≤8	100%
Total cemented surface area ≤144.4 cm^2^	100%
PMMA used ≥60 grams	
Sum of femoral + tibial sizes ≥16	100%
Total cemented surface area ≥158.8 cm^2^	100%

Similarly, in 100% of cases with fem + tib ≥16 (surface area ≥158.8 cm^2^), PMMA used was ≥60-g (Table 5). Finally, for PMMA used ≥60-g, mean fem + tib was 14 (95% CLM 12-17) and mean surface area was 151 cm^2^ (95% CLM 135-167) (Fig. 5).

## Discussion

It has been estimated that the healthcare system in the United States produces nearly 4 million tons of solid waste annually, and operating room waste accounts for more than 30% of hospital waste [7,8]. Notably, a 2020 study reported that within orthopaedic surgery, arthroplasty produced a greater amount of nonrecyclable waste per case than other subspecialties [9].

This is the first study to evaluate PMMA waste during routine TKA, to our knowledge. Our study used 2 × 40 g packages of cement and was restricted to only one brand of MV cement. A recent poll of American Association of Hip and Knee Surgeons members showed that 76.1% of respondents routinely use 2 bags of cement; however, only 36% used MV cement. Our cement technique was similar to that described by the majority of respondents [6].

PMMA waste observed for the 2 surgeons in this series averaged 64 g/59% per TKA. While on a per case basis this may not seem significant, if scaled to larger case volumes, it becomes more relevant. Table 6 demonstrates the waste projected when the observed amounts are scaled to hospital and health system case volumes. In addition, if scaled to 500K cases, up to 35 tons of PMMA waste would potentially be generated annually in the United States alone. Admittedly, these estimates are based on very limited data and should be interpreted with caution. However, if validated by other studies, the potential scale of this waste warrants means to reduce it. Regardless, a focus should remain on 59% average waste, which would not likely be tolerated in any other industry.

**Table 6:** PMMA waste and cost scaled to case volumes.

(Median PMMA waste mass = 64 g/TKA) (median PMMA waste cost = $129/case plain, $154/case gentamicin)		
Volume	Waste g/lb	Waste $ (plain/gentamicin)
Hospital (100 cases/Y)	6400/14.11	$12,900/15,400
Health system (1K cases/Y)	64,000/141.1	$129,000/154,000
National (500K cases/Y)	32M/70.5K (35.3 tons)	$64.5 M/77 M

In this study, the median cost of PMMA waste was $129/TKA overall and increased to $154/TKA when gentamicin cement was used. In this study, the cost of gentamicin cement was 2.5X that of plain PMMA. Table 6 demonstrates that the cost of PMMA waste, scaled to 500,000 cases, could amount to more than $60 million annually.

These results highlight, in particular, the cost of antibiotic cement, the use of which is common in TKA. In a recent poll, 48.2% of respondents reported they “always” or “commonly” used it, the majority of whom reported using commercially prepared antibiotic cement. While advocates for the routine use of antibiotic cement argue its cost benefits if periprosthetic infection is prevented, in the era of bundled payments, any avoidable added costs are detrimental. At 2.5X the cost of plain cement, there would clearly be a benefit in reducing the waste of antibiotic cement in TKA.

We hypothesized that PMMA used would correlate with patient metrics, specifically height and weight, as well as implant size and surface area. However, we observed only low linear correlations (Pearson coefficient) between those metrics and PMMA used. We observed ranges of fem + tib/surface area around clusters of PMMA used. Specifically, 100% of cases with fem + tib ≤8 (surface area ≤144.4 cm^2)^ used ≤40 g PMMA, which could be provided by a single bag of powder. At the opposite extreme, 100% of cases with fem + tib ≥16 (surface area ≥158.8 cm^2^) used ≥60 g PMMA. In these cases, more than one 40 g bag of powder would be required.

Between these 2 ranges, PMMA used varied. In some cases, a single 40 g bag may have sufficed, while others may have required additional powder to prevent a shortfall. One consideration in these cases would be the addition of a 20 g bag of powder, which would add approximately 28 g PMMA. There are disadvantages to this. First, we found that 20 g bags of plain powder were not as readily available as 40 g, and we were unable to obtain any 20 g bags of gentamicin powder. This may be due either to supply issues or the distributor’s preference to market the larger bags, or both. In addition, the pricing of 20 g bags in this study was not favorable. A 20 g bag of plain powder produces PMMA at a cost of $1.23/g, compared to $0.94/g for 40 g plain. The difference for 20 g gentamicin powder is even greater: estimated at $4.38/g PMMA, compared to $2.68/g for 40 g. Finally, the addition of 20 g bags increases inventory and raises the possibility of confusion by circulating staff, who may mistakenly open a 20 g bag instead of a 40 g given overall similarity in packaging. Given the disadvantages of unfavorable pricing and added inventory, we do not recommend routine utilization of 20 g bags of powder.

Though we included patellar size and surface area in our data collection and surface area calculations, we did not specifically evaluate its effect on results. This was for 2 reasons. First, the cemented surface area of the patella represents a small contribution overall, ranging from 8.8% of surface area in the largest constructs in this series to 10.7% in the smallest constructs. In addition, patellar resurfacing is less frequent worldwide [6,[10], [11], [12]] than in the United States, where it is also decreasing [13]. Thus, the frequency and magnitude of patellar cementing make it a very small contributor to cement usage overall.

Though a novel topic, this study admittedly has limitations. We used an indirect method to calculate PMMA used and PMMA waste percent. We chose not to weigh the cannisters pre- and post-cementing because it would interfere with the flow of surgery and could risk contamination. The limitation in this technique is that collection of cement is sometimes incomplete if the cement has hardened within the mixing system or if extruded cement has fallen off the operating field. This bias toward underestimation of cement waste.

Although there was no difference in patient metrics or implant sizes, we observed significant variation in the amount of waste between the 2 surgeons. The difference in mean waste between MD 1 and MD 2 was 23 g. This may have resulted from subtle differences in the standardized cement technique and/or differences in thoroughness of cement retrieval from the cannisters and tubing. While there may have been some differences in cement technique, it was beyond the scope of this study to compare cement mantles postoperatively. Due to differences in retrieval, as discussed, errors in collection would bias toward underestimation of waste.

Though our study was limited to single-manufacturer, MV cement, a recent basic science study found that application of cement in the early doughy phase, as was done in this study, was more important for cement penetration than cement viscosity or manufacturer [13]. This study was restricted to a single implant; however, the inclusion of total cemented surface area may allow our findings of cement requirements to be applied to other implant systems.

Our cost data were generated from contract pricing for 2 large Western US healthcare systems and may not reflect pricing elsewhere. While the universal use of antibiotic cement by one surgeon may have increased the observed costs, it is clear from the recent poll [6] that this is not an unusual practice. In addition, the study’s basic science arm clearly quantitates the much greater cost of antibiotic cement.

**Figure 6:**
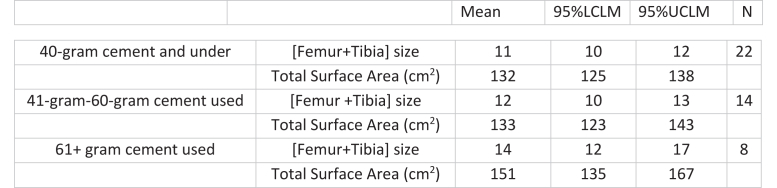
100% Observations of implant size/surface area vs PMMA used.

Finally, it must be acknowledged that zero-waste in cementing is neither practical nor desirable. Some waste is inherent, as the surgeon must ensure an adequate cement mantle for implant fixation, and cement is extruded during compression. However, the fact that more than half the prepared cement is routinely wasted indicates there is room for improvement. An obvious concern would be that in an effort to reduce waste, under-preparation of cement would lead to a shortfall, and a second batch must be prepared. This would lengthen operating time, with its associated risks and costs [[14], [15], [16]]. For those interested in reducing waste, it is recommended they begin trying a single 40 g bag for smaller constructs; as the data showed for fem + tib ≤8 (surface area ≤144.4 cm^2^), this sufficed 100% of the time, with extra cement remaining for margin. In contrast, to prevent shortfall, it is recommended that larger constructs (fem + tib ≥16/ (surface area ≥158.8 cm^2^) always be cemented with 2 × 40 g bags. Between these 2 extremes, the 95% CLM (Figure 5, Figure 6) shows ranges where single 40 g bags may suffice. A surgeon who has become comfortable cementing smaller constructs with a single bag could potentially expand into these ranges and find their personal “comfort level” with using a single bag.

This study is small and must be validated by others with a wider variety of implants, cement techniques, manufacturers, and viscosities. However, as a pilot study, it may stimulate more efficient use of PMMA and reduce waste, resulting in both cost savings and improved sustainability within orthopaedic surgery.

## Conclusions

This prospective study found that when two 40 g bags of PMMA are routinely mixed for primary TKA, on average more than 50% of the produced PMMA is discarded as waste, at a median cost of $129/case. Scaled to national case volume (500K), this would produce up to 35 tons of PMMA waste annually at a cost of more than $60 million. PMMA waste may be reduced by surgeons evaluating their own use of cement, including correlating implant sizes to expected PMMA requirements. Any reduction in waste will benefit the environment by reducing landfill deposits and reducing costs. While a goal of zero cement waste is neither practical nor desirable, any reasonable reduction of PMMA waste would be both environmentally and financially beneficial.

## Figures and Tables

**Conflict of Interest Statement for Tompkins:**
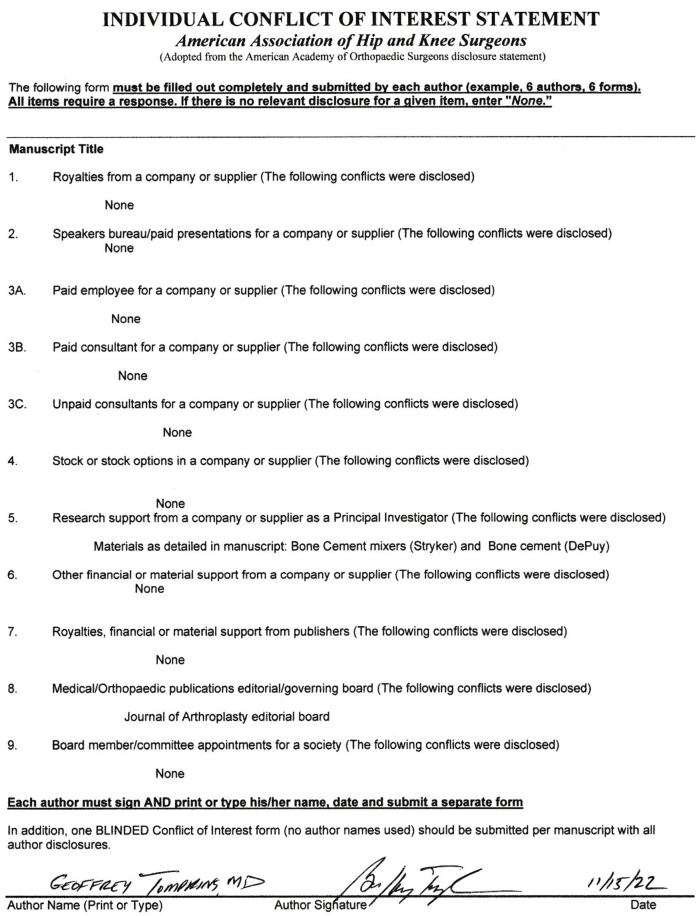


## Appendix A Supplementary data

Conflict of Interest Statement for Sypher

Conflict of Interest Statement for Chiu

Conflict of Interest Statement for Valderrama

Conflict of Interest Statement for Howe

Conflict of Interest Statement for Griffin
