# Supplementary material for: Bone Cement Waste in Total Knee Arthroplasty: A Preliminary Study of Scope and Cost
Source: Arthroplast Today. 2023 Jul 24;22:101162. doi: 10.1016/j.artd.2023.101162 (PMC10393734; doi:10.1016/j.artd.2023.101162)
Supplement: Conflict of Interest Statement for Chiu [file mmc2.pdf]

INDIVIDUAL CONFLICT OF INTEREST STATEMENT

American Association of Hip and Knee Surgeons

(Adopted from the American Academy of Orthopaedic Surgeons disclosure statement)

The following form **must be filled out completely and submitted by each author (example, 6 authors, 6 forms). All items require a response. If there is no relevant disclosure for a given item, enter "*None*."**

Manuscript Title

1. Royalties from a company or supplier (The following conflicts were disclosed)

   No conflicts.

2. Speakers bureau/paid presentations for a company or supplier (The following conflicts were disclosed)

   No conflicts.

3A. Paid employee for a company or supplier (The following conflicts were disclosed)

   No conflicts.

3B. Paid consultant for a company or supplier (The following conflicts were disclosed)

   No conflicts.

3C. Unpaid consultants for a company or supplier (The following conflicts were disclosed)

   No conflicts.

4. Stock or stock options in a company or supplier (The following conflicts were disclosed)

   No conflicts.

5. Research support from a company or supplier as a Principal Investigator (The following conflicts were disclosed)

   No conflicts.

6. Other financial or material support from a company or supplier (The following conflicts were disclosed)

   No conflicts.

7. Royalties, financial or material support from publishers (The following conflicts were disclosed)

   No conflicts.

8. Medical/Orthopaedic publications editorial/governing board (The following conflicts were disclosed)

   No conflicts.

9. Board member/committee appointments for a society (The following conflicts were disclosed)

   No conflicts.

Each author must sign AND print or type his/her name, date and submit a separate form

In addition, one BLINDED Conflict of Interest form (no author names used) should be submitted per manuscript with all author disclosures.

Shih-Ting Chiu — ShihTing Chiu — 11/16/2022

Author Name (Print or Type) — Author Signature — Date
